# Controllable Preparation and Filtration Performance of New Composite Materials for Mining Masks

**DOI:** 10.3390/ma19030626

**Published:** 2026-02-06

**Authors:** Xin Zhang, Chenyu Zhang, Tianyu Zhou, Zeyu Sun, Yong Jin, Lihua Mi, Saisai Wu

**Affiliations:** 1 College of Architecture and Energy Engineering, Wenzhou University of Technology, Wenzhou 325000, China; 2 School of Resources Engineering, Xi′an University of Architecture and Technology, Xi’an 710055, China; 3 School of Building Services Science and Engineering, Xi′an University of Architecture and Technology, Xi’an 710055, China

**Keywords:** mining mask, metal mines, work intensity, filtration efficiency, performance

## Abstract

The effective isolation of high-concentration dust during mining and transportation processes is a hot issue of occupational health concern for workers. Based on the characteristics of graphene and its derivatives, a new type of mining mask made of graphene oxide polypropylene composite material (GO polypropylene composite material) and reduced graphene oxide polypropylene composite material (rGO polypropylene composite material) was prepared using the direct impregnation method. Moreover, particle filtration performance tests were conducted under different gas flow conditions. The results showed that, at the same concentration, the reduction group (rGO polypropylene composite material) had more compliance indicators, and the comprehensive performance ranking was as follows: reduction group (rGO polypropylene composite material) > oxidation group (GO polypropylene composite material) > control group (polypropylene composite material). The reduction group with a concentration of 0.3 g/L showed the best overall performance. At a flow rate of 1.0 m^3^/h, the filtration efficiency of PM_10_ (95.61%) and PM_2.5_ (95.01%) met the relevant standards, while PM_1.0_ (94.88%) was close to the standard threshold (with a difference of only −0.12%), significantly better than the control (PM_10_, 93.39%) and the oxidation (PM_10_, 95.01%) groups. Moreover, at various flow rates, its particulate matter concentration was significantly lower than that for the oxidation and control groups. Overall, it meets the requirements of providing ideal filtration effects under different work intensities (low, medium, and high flow rates), thus providing strong technical support for individual protection of mine workers and a theoretical basis and practical guidance for reducing occupational diseases caused by dust exposure in mines.

## 1. Introduction

Mineral resources, as the cornerstone of the global industrial system, are continuously expanding in scale with the industrialization process [1]. However, with the continuous deepening of mining operations, the amount of dust generated during the operation process increases rapidly, seriously polluting the working environment, threatening the health of employees, and potentially even causing explosions [2,3]. The main sources of dust during the mining process are drilling, blasting, loading, unloading, and transportation [4]. A large amount of respiratory dust, toxic and harmful gases, and corrosive aerosols enter the human body through respiration and can cause serious occupational diseases such as pneumoconiosis, chronic bronchitis, and poisoning [5,6]. As of the end of 2022, a total of 915,000 occupational patients had been reported nationwide, of which 90% had occupational pneumoconiosis, mainly distributed in the mining industry [7]. Pneumoconiosis in miners is known as the “invisible killer” that endangers their physical health. Only by blocking the inhalation of dust can pneumoconiosis be prevented [8]. Therefore, improving the performance of mining respiratory protection equipment is urgently needed to ensure the life and health of miners and promote the green and safe development of mining.

Personal protective equipment mainly serves the dual functions of droplet barrier and dust filtration [9], with special usage scenarios imposing far more stringent requirements on mask materials than on ordinary civilian masks. However, the self-priming masks widely used in mines currently have significantly reduced protective effectiveness in operating environments with severely excessive dust concentration and cannot provide stable and effective protective performance. In particular, the labor intensity is relatively high for frontline mine workers, leading to an increase in respiratory flow. As the amount of dust filtered increases, the respiratory resistance of the filter cotton also increases, affecting work efficiency and increasing the probability of workers suffering from pneumoconiosis [10]. At the same time, masks need to balance comfort and durability to avoid increased usage costs and operational interruption risks due to frequent replacement [11]. In recent years, an increasing number of scholars have studied the filtration efficiency of masks [12,13,14,15,16], which have evolved from natural fibers such as cotton and linen to glass fibers and polypropylene meltblown fabrics as the core, and are currently developing in composite, functional, and intelligent directions [17,18]. At present, research mainly focuses on the protective performance of different masks [11,12], the development of new masks [13], the application of PTFE-coated masks [14], and the filtration mechanism of masks [15]. Although some research success has been achieved, there is still insufficient research on their performance in special places such as mines; for example, the problem of heat damage becomes more prominent with the increasing depth of mines [19], and the particularity of their underground environment is also significantly different from the daily surface environment. The performance requirements of protective masks need to be continuously improved to meet the actual needs of engineering.

The main practical issues with existing composite materials for mining masks include the following [20,21,22,23]. First, there is a trade-off between filtration efficiency and breathability: traditional materials often enhance filtration efficiency by reducing fiber pore size and increasing filter layer thickness, leading to significantly higher airflow resistance. Prolonged use by miners can cause discomfort such as chest tightness and shortness of breath, and may even lead to unauthorized mask removal due to breathing difficulties, thereby increasing safety risks [20,21]. Second is insufficient moisture resistance: most filtration materials have high surface hydrophilicity, making them prone to moisture absorption and caking in the high-humidity underground environment. This results in rapid pore blockage and a sharp decline in filtration efficiency, failing to meet the demands of prolonged operations [22]. Third is weak synergistic protective capabilities: existing materials primarily focus on single pollutant filtration, either filtering dust or adsorbing only small amounts of toxic gases. Additionally, these materials exhibit low mechanical strength, are prone to damage, and have relatively short lifespans. Frequent replacement not only raises economic costs but also generates substantial medical waste, contradicting the principles of green environmental protection [23]. Research on protective masks for underground metal mines still requires continuous investigation.

To this end, novel composite protective masks have become a current research hotspot. Graphene, a new nanomaterial, has been widely applied in various fields due to its unique physicochemical properties [24]. In recent years, graphene and its derivatives (such as graphene oxide and reduced graphene oxide) have demonstrated innovative potential in the field of mine air purification materials [25]. The combined effects of multiple pollutants in mine environments have led to issues such as rapid filtration efficiency decay and insufficient chemical stability in traditional filter materials. The excellent mechanical properties, chemical stability, and high adsorption capacity of graphene materials make them an ideal choice for mine air filtration materials [26]. Their abundant active sites can be functionalized to achieve adsorption and catalytic degradation of toxic gases [27], while the water contact angle is increased to over 120°, significantly enhancing wet resistance [28]. Additionally, the internal micro-nano channels can efficiently capture fine dust particles through mechanisms such as “interception, inertial collision, and diffusion” while also enabling rapid airflow passage, significantly improving filtration efficiency [29]. With the continuous advancement of graphene technology, its applications in mine air purification will become more widespread. However, research on masks made from composite polypropylene (PP) materials using graphene and its derivatives remains relatively limited, with even less investigation into the filtration performance of these novel composite masks. Comprehensive studies on the purification of mine pollutants by these masks are nearly nonexistent.

Therefore, in response to the aforementioned practical conditions, this study developed and prepared a highly efficient and practical composite filter material suitable for use in underground metal mines. The newly developed composite filter material was experimentally tested under varying flow rates and operational conditions. By analyzing the patterns of filtration performance changes, this research promotes the optimization of protective measures, provides reliable guidance for selecting protective masks for mine workers, and ensures safe and efficient mine operations.

## 2. Methods

### 2.1. Performance Parameters: Selection of Materials

The experimental materials include polypropylene material, produced by Guangdong Fresh Filter Co., Ltd., Foshan, China, and the industrial-grade single-layer graphene powder, produced by Suzhou Tan Feng Graphene Technology Co., Ltd., Suzhou, China, with a purity of 0.95. The reducing agent used was L-ascorbic acid, produced by Tianjin Kemio Chemical Reagent Co., Ltd., Tianjin, China, and analyzed as pure AR. The deionized water was produced by Hangzhou Yongjieda Purification Technology Co., Ltd., Hangzhou, China, with a pure water machine.

Graphene oxide polypropylene composite material (GO polypropylene composite material) and reduced graphene oxide polypropylene composite material (rGO polypropylene composite material) were prepared through graphene modification of polypropylene materials. The modification process is as follows: degreasing → drying → preparing impregnation solution → ultrasound → water bath impregnation → drying → preparing reducing agent → reduction → drying. The size is 5 cm × 5 cm, with the first block as the blank group, the second block as the oxidation group, and the third block as the reduction group.

The detailed process of preparing GO–rGO polypropylene composite material is as follows:

Immerse the 5 cm × 5 cm PP samples in a neutral detergent solution or ethanol (volume fraction 75%) for 15–20 min. After cleaning, rinse the samples thoroughly with deionized water 3–5 times to eliminate detergent or ethanol residues. Place the degreased PP samples in a vacuum drying oven. Dry at 70 °C for 2 h. The following steps are adopted to prepare impregnation solutions with different concentrations (0.2 g/L, 0.3 g/L, 0.4 g/L, and 0.5 g/L), as required by the experiment. Perform ultrasonic treatment at 200 W power for 60 min, with 5 s on/2 s off intervals to avoid excessive temperature rise. The final solution should be homogeneous and free of obvious sedimentation. Immerse the dried PP samples vertically into the prepared graphene dispersion. Place the beaker containing the solution and samples in a constant temperature water bath. Maintain the temperature at 60 °C and impregnate for 2 h. Blot the excess solution on the surface with filter paper, then place the samples in a vacuum drying oven. Dry at 60 °C for 2 h under vacuum to remove the solvent (deionized water) and obtain oxidation group samples (GO-PP composites). Dissolve L-ascorbic acid in deionized water to prepare a 0.1 mol/L solution. Immerse the GO-PP composites into the L-ascorbic acid solution. Place the mixture in a constant temperature water bath at 70 °C to react for 2 h. The solution gradually changes from light yellow to black, indicating the progress of the reduction reaction. Take out the samples after the reaction and rinse them with deionized water 3–5 times to remove unreacted L-ascorbic acid and reaction by-products. Then, dry the samples in a vacuum drying oven at 60 °C for 2 h to obtain reduction group samples (rGO-PP composites).

### 2.2. Experimental Systems

**Figure 1:**
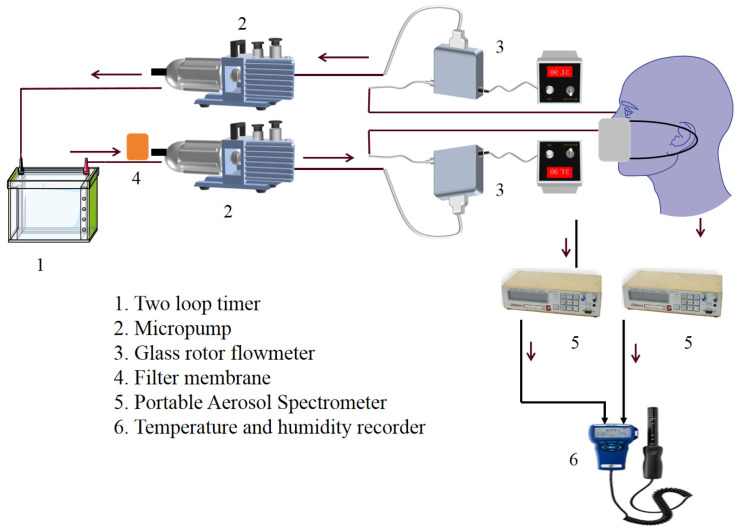
Experimental setup.

We designed a dual-channel timing control module to drive the micro pump group to operate alternately and constructed a respiratory dynamic simulation device to simulate and reproduce the dynamic process of human respiration. The working principle of the model experimental device is shown in Figure 1.

In the experiment, different concentrations of graphene solutions (0.2 g/L, 0.3 g/L, 0.4 g/L, and 0.5 g/L) were used to prepare the new filter material, resulting in the formation of corresponding filter membranes. A glass rotor flowmeter was set up to accurately control the flow rate of gas, such that the flow state of gas could be stable under preset conditions, ensuring the reliability of the experimental results. In the experiment, each breathing cycle lasted for 4 s. Ventilation was simulated and stopped for 1 s to allow the gas to mix in the pipeline. Three sets of materials were tested three times each, with a total of 36 experiments conducted at four concentrations. The average of repeated test data was taken for analysis, such that the results could better reflect the actual effect. The GRIMM1.109 Portable Aerosol Spectrometer was used to test the mass concentration of particulate matter, supplied by Beijing Saak-Mar Environmental Instrument Ltd., Beijing, China. The upper limit of concentration that could be counted was 2,000,000 particles per liter (P/L). The measurement range was 0.1~100,000 μg/m^3^. The repeatability was 5%.

### 2.3. Performance Parameters

This experiment uses a glass rotor flowmeter to stabilize the gas flow rates to 1.0 m^3^/h, 1.5 m^3^/h, and 2.0 m^3^/h to simulate the respiratory intensity of the human body under mild, moderate, and severe labor intensity [30]. This helped us to analyze the actual performance of the new mask filter material under different working intensities.

The filtration efficiency was calculated using Equation (1) [31]:

(1) $$\eta =\frac{C_{1}-C_{2}}{C_{1}}\times 100\%$$

where *η* is the filtration efficiency (%), *C*_1_ is the concentration of particulate matter before filtration (μg/m^3^), and *C*_2_ is the concentration of particulate matter after filtration (μg/m^3^).

## 3. Results

### 3.1. Particle Concentration and Particle Size Distribution

**Figure 2:**
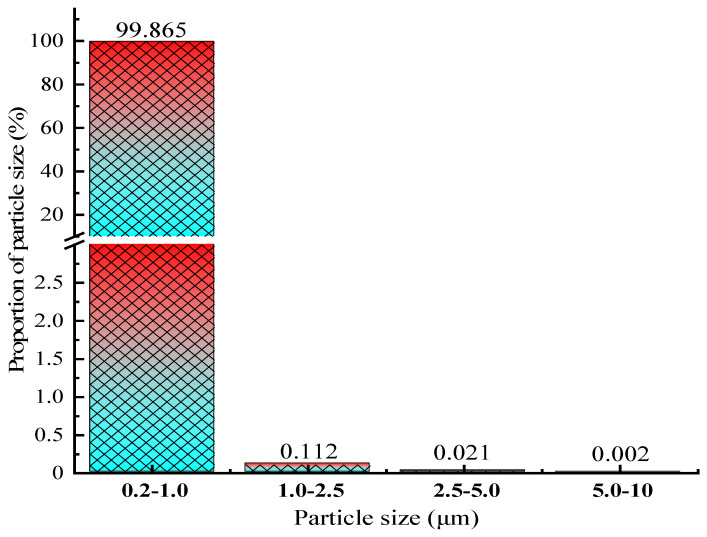
Particle size distribution.

The particle size distribution of dust in the atmosphere was obtained by systematically measuring the concentration of particulate matter in the testing environment, as shown in Figure 2.

Figure 2 shows that, under the test environment parameters (the average temperature is 19.9–25.5 °C, and the average humidity is 38.9–50.9%), the majority of particles have a diameter of 0.2–2.5 μm, accounting for about 99.98%, with particles with a diameter of 0.2–1.0 μm accounting for 99.87%. Obvious unimodal distribution characteristics were observed in the particle size range of 0.2–1.0 μm, which is significantly consistent with the typical particle size distribution pattern formed by homogeneous nucleation condensation growth of secondary aerosols [32]. Coarse particulate matter (>1 μm) in the atmosphere exhibits significant gravitational settling effects, resulting in shorter residence times and limited diffusion ranges. Under the random thermal motion of air molecules, particles with a diameter of less than 1 μm exhibit Brownian diffusion characteristics, resulting in a significant decrease in their settling velocity. They can maintain a suspended state for a long time and be widely distributed in space, leading to fine particles dominating the aerosol system in this environment [33].

According to epidemiological research data from the World Health Organization (WHO), there is a significant exposure response relationship between the daily mass concentration of PM_10_ and population health risks: for every 10 μg/m^3^ increase in concentration, the relative risk of all-cause mortality increases by 0.5%; when the exposure level reaches the critical threshold of 50 μg/m^3^, the risk factor will climb to 5% [34]. Based on the principle of minimizing health risks, this test selected the first-level limit value of China’s “Ambient air quality standards” (GB 3095-2012) [35] as the benchmark, which stipulates that the 24 h average concentration control values for PM_2.5_ and PM_10_ are 35 μg/m^3^ and 50 μg/m^3^, respectively. By establishing a particle size segmented mass concentration distribution model, the quantitative results show that the 24 h mean values of PM_1.0_, PM_2.5_, and PM_10_ are 14.78 μg/m^3^, 15.97 μg/m^3^, and 18.36 μg/m^3^, respectively. From the characteristics of fine particulate matter pollution, the mass concentration ratio of PM_2.5_/PM_10_ is 0.87, indicating that the inhalable particulate matter in the monitoring environment is mainly composed of fine particles.

### 3.2. Filtration Efficiency Results and Analysis

#### 3.2.1. Filtration Efficiency at a Gas Flow Rate of 1.0 m^3^/h

**Figure 3:**
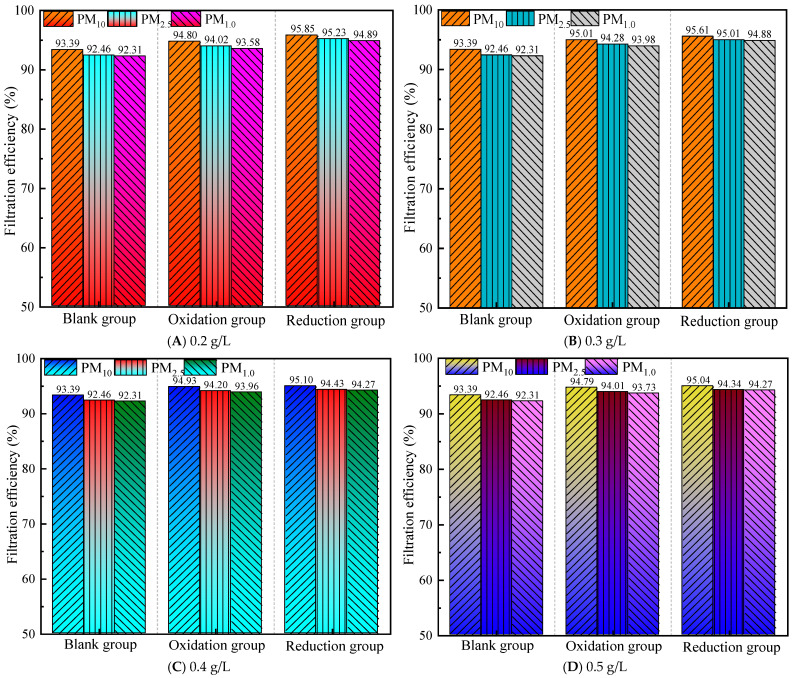
Filtration efficiency at a gas flow rate of 1.0 m^3^/h.

According to the national standard GB/T 32610-2016, Technical specification of daily protective mask [36], and GB2626-2019, Respiratory protection—Non-powered air-purifying particle respirator [37], protective materials in industrial sites should achieve a filtration efficiency of ≥95% for both KN95 and N95 categories. As shown in Figure 3, the difference between the average filtration efficiency of the control group for PM_10_ and the standard is −1.61%, for PM_2.5_, it is −2.54%, and for PM_1.0_, it is −2.69%, with a range of standard deviation values from −2.69% to −1.61%. This indicates that the control group has significantly insufficient filtering ability for all three types of particulate matter, especially for PM_1.0_, which has the greatest filtering defect.

After graphene modification, at a gas flow rate of 1.0 m^3^/h, all concentrations (0.2~0.5 g/L) of the modified group were significantly better than those of the control group, indicating that graphene treatment can improve filtration performance, and the reduction group performed better than the oxidation group. At the same concentration, the reduction group achieved more standard indicators. The comprehensive performance ranking is reduction group > oxidation group > control group.

For example, at 0.2 g/L, the reduction group meets the PM_10_/PM_2.5_ standard, while the oxidation group does not meet the standard at all. All experimental groups showed that PM_1.0_ filtration was the weakest, with a consistently negative difference (maximum: −1.42%), indicating that the existing modification scheme still needs to improve its ability to intercept fine particles. PM_10_ has the highest compliance rate (four times), followed by PM_2.5_ (two times), which conforms to the rule that the smaller the particle size, the more difficult it is to filter. The reduction group achieved PM_10_/PM_2.5_ compliance at 0.2 g/L, and the PM_10_ difference reached the maximum positive value (+0.85%), indicating that low-concentration reduction treatment can effectively improve coarse particle filtration.

At a concentration of 0.3 g/L, the overall performance was the best, with the reduction group meeting the standards for PM_10_ and PM_2.5_, and PM_1.0_ approaching the standards (difference: −0.12%). The oxidation group met the standards for PM_10_ for the first time, but there were still differences in other particulate matter.

At a concentration of 0.4–0.5 g/L, the filtration efficiency of the oxidation group’s PM_10_ decreased compared to 0.3 g/L (from 95.01% to 94.93% to 94.79%), possibly due to material accumulation leading to a decrease in porosity.

At a gas flow rate of 1.0 m^3^/h, selecting the 0.3 g/L reduction group scheme as the optimal parameter can simultaneously meet the PM_10_/PM_2.5_ standard requirements and approach the PM_1.0_ compliance threshold.

#### 3.2.2. Filtration Efficiency at a Gas Flow Rate of 1.5 m^3^/h

**Figure 4:**
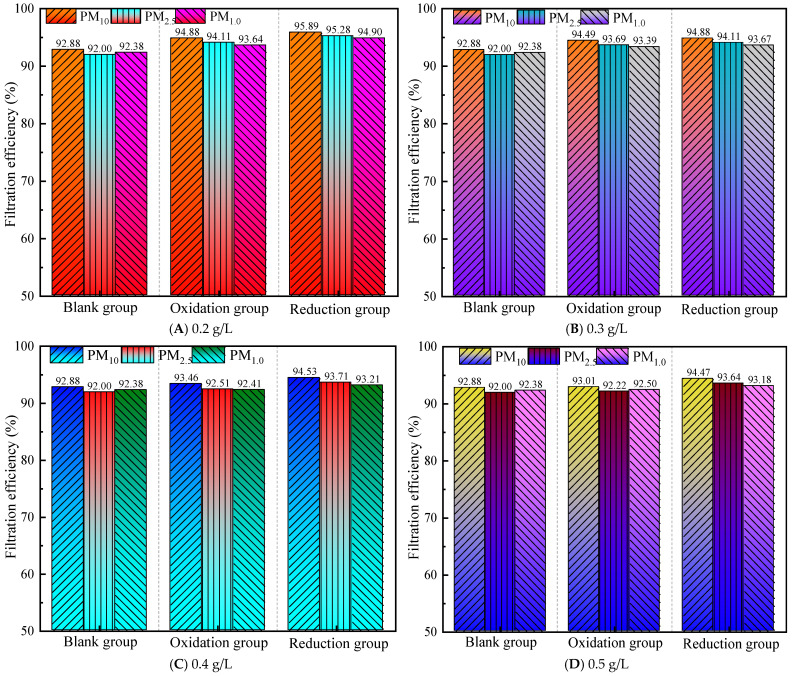
Filtration efficiency at a gas flow rate of 1.5 m^3^/h.

Figure 4 shows that compared to 1.0 m^3^/h, the filtration efficiency of all groups significantly decreased at a flow rate of 1.5 m^3^/h. The efficiency of the control group further decreased at 1.5 m^3^/h compared to 1.0 m^3^/h, and the difference range expanded to −3~−2.12%, indicating that the basic filtration system is more sensitive to flow changes. The modification effect of graphene shows that, at a gas flow rate of 1.5 m^3^/h, the comprehensive performance ranking is reduction group > oxidation group > control group. All groups showed a decreasing efficiency trend of PM_10_ > PM_2.5_ > PM_1.0_, with the difference range expanding to −1.82% to 0.89%. At a flow rate of 1.5 m^3^/h, the efficiency of PM_1.0_ further decreased compared to 1.0 m^3^/h, with the difference widening to −1.82%, indicating that high flow rates have a more significant negative impact on ultrafine particle filtration.

The reduction group performed well at low concentrations of 0.2–0.3 g/L, showing the best performance at a concentration of 0.2 g/L. However, further optimization was needed for PM_1.0_, as both PM_10_ and PM_2.5_ met the standard, and PM_1.0_ was close to the standard (difference: −0.10%). However, the efficiency of the oxidation group and high-concentration scheme significantly decreased, indicating that low-concentration reduction treatment can still maintain high efficiency at high flow rates. Increasing the flow rate has a significant negative impact on filtration efficiency, especially weakening the filtration capacity of PM_1.0_. The efficiency of the oxidation and reduction groups significantly decreased at 0.4–0.5 g/L; in particular, the PM_10_ efficiency of the oxidation group decreased from 94.88% (0.2 g/L) to 93.01% (0.5 g/L), indicating that high concentrations lead to material aggregation or pore blockage, resulting in a decrease in filtration efficiency [38], which is detrimental to performance.

#### 3.2.3. Filtration Efficiency at a Gas Flow Rate of 2.0 m^3^/h

**Figure 5:**
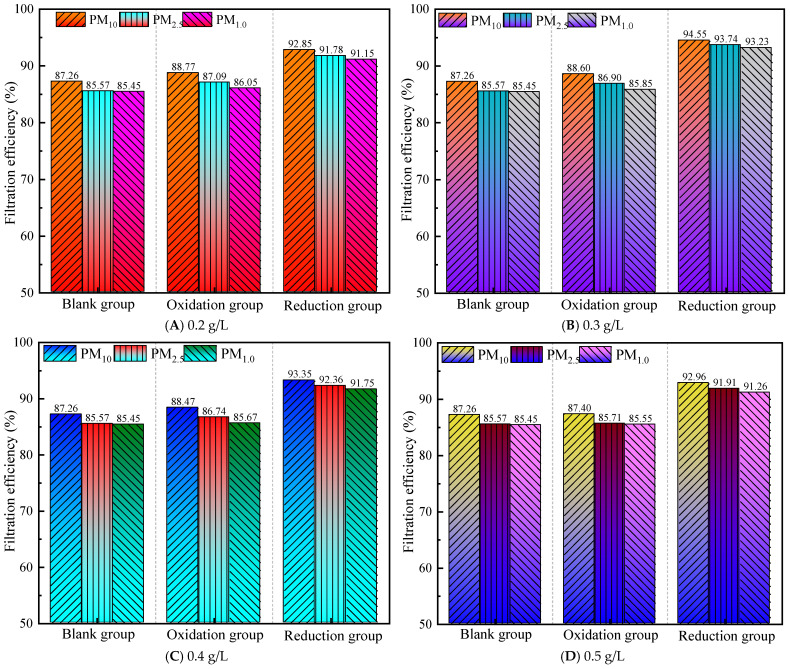
Filtration efficiency at a gas flow rate of 2.0 m^3^/h.

As shown in Figure 5, compared to flow rates of 1.0 m^3^/h and 1.5 m^3^/h, the efficiency of all groups further decreased at 2.0 m^3^/h. In the control group, the PM_10_ efficiency decreased from 93.39% (1.0 m^3^/h) to 87.26%, and the difference range expanded to −9.55~−7.74%. In the reduction group, the PM_10_ efficiency decreased from 95.85% (1.0 m^3^/h) to 94.55% (0.3 g/L), and the difference shifted from meeting the standard to approaching the standard (−0.45%). The efficiency of the PM_2.5_ and PM_1.0_ reduction groups increased by 5% to 7% compared to the oxidation group, and all groups showed an efficiency ranking of PM_10_ > PM_2.5_ > PM_1.0_, with the difference range expanding to −9.55% to −0.45%, confirming that particles with small particle size are more likely to penetrate the filter medium. At the same time, the difference in PM_1.0_ is further expanded. PM_1.0_ is dominated by Brownian motion, and traditional mechanical filtration mechanisms have low efficiency, requiring the introduction of electrostatic adsorption or chemical bonding mechanisms [39].

At a gas flow rate of 2.0 m^3^/h, the 0.3 g/L reduction group is the optimal solution, with PM_10_, PM_2.5_, and PM_1.0_ efficiencies close to the standard and showing strong adaptability to flow rate changes, indicating that this concentration may balance material loading and porosity. The high-concentration graphene scheme (≥0.4 g/L) resulted in a significant decrease in efficiency due to pore blockage. For example, the PM_1.0_ difference in the 0.5 g/L reduction group increased from −1.82% (1.5 m^3^/h) to −3.74% (2.0 m^3^/h), indicating that the synergistic negative effects of high concentration and high flow rate are significant and should be avoided.

### 3.3. Counting Concentration Results and Analysis

#### 3.3.1. Particle Concentration After Filtration at 0.2 g/L

**Figure 6:**
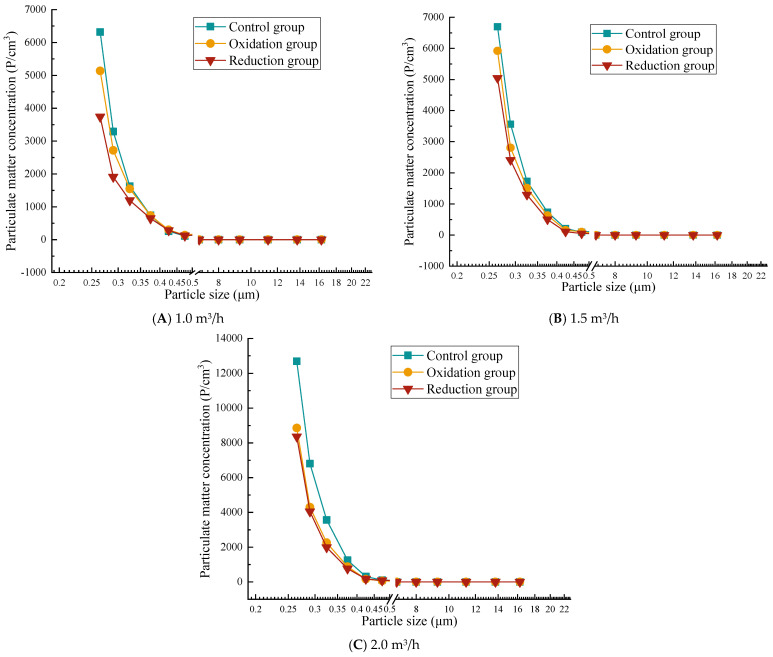
Particle concentration after filtration at 0.2 g/L.

Figure 6 shows that the overall trend of particle concentration increases with the increase in flow rate, but the particles with a diameter of less than 1.0 μm still have higher concentrations, among which the concentration of particles with a diameter of 0.265–0.475 μm changes rapidly. The particle concentration in the reduction group was significantly lower than that in the oxidation group and the control group. The efficiency of the reduction group significantly decreased at a high flow rate of 2.0 m^3^/h. A higher respiratory rate led to a significantly increased concentration of small particles. The reduction group significantly reduced the concentration of large particles at all flow rates, while the concentration of large particles in the control group and oxidation group increased with increasing respiratory rate. At a low flow rate of 1.0 m^3^/h, the concentration range of particulate matter is relatively small (up to about 6500 P/cm^3^). The concentration of the control and oxidation groups is evenly distributed in the particle size range of 1–10 μm, while the concentration of the reduction group significantly decreases at larger particle sizes (10 μm). At a medium flow rate of 1.5 m^3^/h, the concentration range of particulate matter expands (to about 7000 P/cm^3^), and the overall concentration distribution is similar to that at low flow rates. At a high flow rate of 2.0 m^3^/h, the concentration significantly increases (up to 13,000 P/cm^3^), indicating that an increase in respiratory rate leads to more suspended particulate matter, especially for small particles below 1 μm, where the concentration increases significantly.

#### 3.3.2. Particle Concentration After Filtration of 0.3 g/L

**Figure 7:**
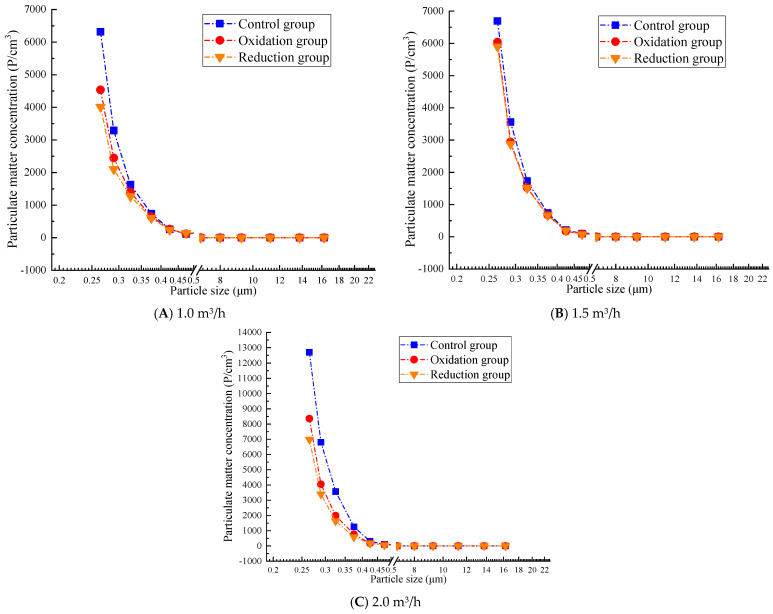
Particle concentration after filtration at 0.3 g/L.

Figure 7 shows that the overall trend of particle concentration in a 0.3 g/L graphene solution increases with increasing flow rate, and the particle penetration rate increases at high flow rates. The particle concentration of the reduction group was significantly lower than that of the oxidation and control groups, which verified the filtration performance of the graphene-modified group: reduction group > oxidation group > control group. At all flow rates, the concentration of particulate matter significantly decreases with increasing particle size. In particular, when the particle size is less than 1.0 μm, the particle quantity concentration is high, while as the particle size increases to 2.0 μm or more, the particle quantity concentration sharply decreases, even approaching zero. At a low flow rate of 1.0 m^3^/h, the concentration range of particulate matter is relatively small (up to about 6500 P/cm^3^). At a medium flow rate of 1.5 m^3^/h, the concentration range of particulate matter expands (to about 7000 P/cm^3^). At a high flow rate of 2.0 m^3^/h, the concentration significantly increases (up to 13000 P/cm^3^), similar to the results for 0.2 g/L concentration. This also confirms that the filtration efficiency test results show that 0.2 g/L and 0.3 g/L concentrations have the best filtration efficiency at low flow rates. However, the 0.3 g/L reduction group performed better than the 0.2 g/L group. At a concentration of 0.3 g/L, the reduction group showed a better interception efficiency of coarse particles (PM_10_) than 0.2 g/L in the flow rate range of 1.0–2.0 m^3^/h. However, there is still a bottleneck for PM_1.0_. At 1.0 m^3^/h, PM_10_ approaches the standard and, at 2.0 m^3^/h, the efficiency is significantly better than 0.2 g/L.

#### 3.3.3. Particle Concentration After Filtration at 0.4 g/L

**Figure 8:**
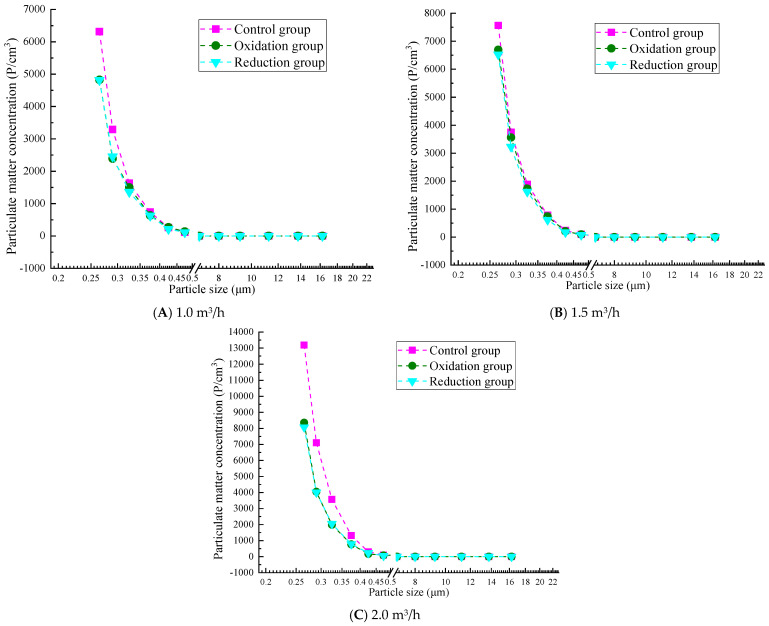
Particle concentration after filtration at 0.4 g/L.

From Figure 8, it can be seen that when the gas flow rate is 1.0 m^3^/h, in smaller particle size ranges (such as 0.265 μm and 0.290 μm), the particle concentration of the control, oxidation, and reduction groups is relatively high, and there is a large difference in concentration with the front end of the atmosphere. As the particle size increases, the number and concentration of particles gradually decrease, especially in the particle size range of 0.615 μm and above, where the number and concentration of particles significantly decrease, indicating that the graphene solution has a better filtration effect on smaller particles at low flow rates. Compared with 1.0 m^3^/h, the particle quantity concentration at a flow rate of 1.5 m^3^/h increased in the smaller particle size range, especially in the particle size ranges of 0.265 μm and 0.290 μm. As the flow rate increases, the number and concentration of particulate matter further decrease in larger particle size ranges (such as 0.615 μm and above), but particulate matter can still be effectively filtered at moderate flow rates. When the gas flow rate is 2.0 m^3^/h, the particle concentration is the highest, and the filtration efficiency is lower at smaller particle sizes. However, as the particle size increases, the filtration efficiency rapidly improves. Compared with the concentrations of 0.2 g/L and 0.3 g/L, it can be seen that at a solution concentration of 0.4 g/L, the range of particulate matter is larger and its filtration efficiency decreases, which is consistent with the results of the weight filtration efficiency test.

#### 3.3.4. Particle Concentration After Filtration at 0.5 g/L

**Figure 9:**
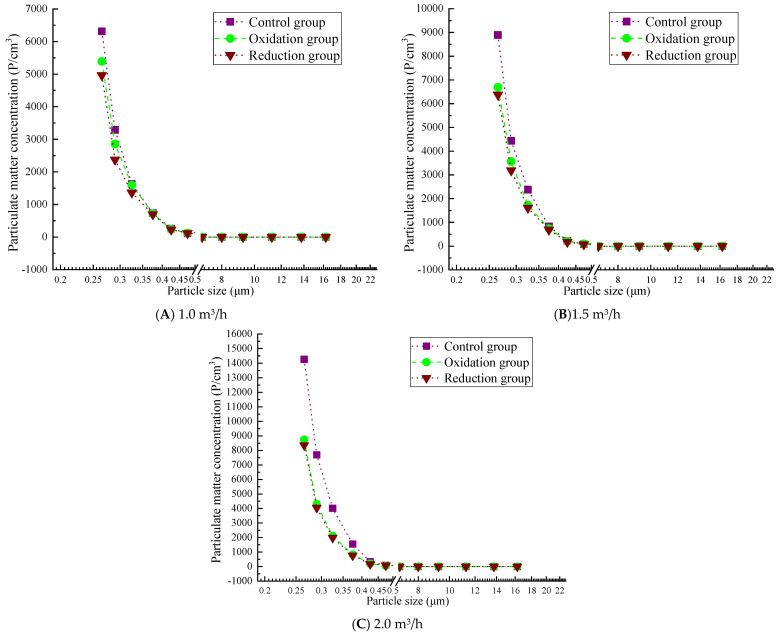
Particle concentration after filtration at 0.5 g/L.

As shown in Figure 9, at a low flow rate of 1.0 m^3^/h, the concentration of large particles (e.g., 0.750–1.800 μm) is relatively high in the reduction group, and the concentration of some particles (e.g., 1.150 μm) shows non-monotonic changes with decreasing flow rates. This is because at low flow rates, the fluid shear force weakens, the probability of particle collision increases, and, under the dominance of van der Waals forces, larger particle size aggregates are more likely to form [40]. This is particularly evident in the reduction group, possibly due to the reduction treatment reducing the surface charge of particles and weakening the electrostatic repulsion effect [41]. At a moderate flow rate of 1.5 m^3^/h, the concentration of small particles in the oxidation and reduction groups is between those at high and low flow rates, but the reduction group shows a significant increase in the number of particles with larger diameters (such as 2.250 μm or larger). At a high flow rate of 2.0 m^3^/h, the average small particle size (such as 0.265–0.425 μm) was significantly higher in the control group and oxidation group than those under low flow rate conditions (1.0 and 1.5 m^3^/h). For example, the concentration of 0.265 μm particles in the control group was 14,260 at 2.0 m^3^/h, but only 6316 at 1.0 m^3^/h. A higher gas flow rate may enhance turbulence and shear forces in the solution, promote physical exfoliation of graphene layers, and generate more small-sized particles.

## 4. Discussion

### 4.1. Comparison and Summary of Filtration Efficiency

As the gas flow rate increased from 1.0 m^3^/h to 2.0 m^3^/h, the filtration efficiency of all groups significantly decreased, with a more pronounced impact on PM_1.0_. The PM_10_ efficiency of the control group dropped from 93.39% (1.0 m^3^/h) to 87.26% (2.0 m^3^/h), and the difference range expanded from −2.69~−1.61% to −9.55~−7.74%. For the reduction group, the PM_10_ efficiency decreased from 95.85% (1.0 m^3^/h) to 94.55% (2.0 m^3^/h), shifting from compliance to near compliance (−0.45%). This was due to the high flow rate shortening the residence time of particles in the filter medium, weakening physical interception and adsorption effects, and more significantly impacting PM_1.0_ capture. At all flow rates and concentrations, the filtration efficiency of the reduction group was higher than that of the oxidation group, particularly under high flow rates of 1.5 m^3^/h and 2.0 m^3^/h. At a gas flow rate of 1.0 m^3^/h, the PM_10_ efficiency of the reduction group reached 95.85% (compliant), while the oxidation group was 94.80% (non-compliant). At a gas flow rate of 2.0 m^3^/h, the PM_10_ efficiency of the reduction group reached 94.55% (near compliant), while the oxidation group was 88.60% (non-compliant). This was attributed to the reduction treatment enhancing the conductivity and surface activity of graphene [42], improving the electrostatic adsorption capacity of particles and performing better under high-speed airflow. A low-concentration graphene solution maintained high filtration efficiency even at high flow rates, while high concentrations (≥0.4 g/L) led to a significant decline due to pore blockage. At a gas flow rate of 1.5 m^3^/h, the PM_10_ efficiency of the 0.2 g/L reduction group reached 95.89% (compliant), whereas it dropped to 94.47% (non-compliant) at 0.5 g/L. At a gas flow rate of 2.0 m^3^/h, the PM_10_ efficiency of the 0.3 g/L reduction group reached 94.55% (near compliant), while it declined to 92.96% (non-compliant) at 0.5 g/L. It can be observed that 0.2 g/L and 0.3 g/L concentrations exhibited optimal filtration efficiency at low flow rates, with a notable decline at high flow rates. Among them, the 0.3 g/L concentration maintained stable filtration efficiency across all flow rates, nearly meeting compliance standards, while the 0.4~0.5 g/L high concentrations showed a significant efficiency drop. After a comprehensive comparison of weight efficiency, the 0.3 g/L reduction group was selected as the optimal concentration solution, balancing efficiency, stability, and cost-effectiveness within the 1.0~2.0 m^3^/h flow rate range. It is suitable for industrial filtration scenarios with dynamic flow variations and demonstrates excellent performance in underground metal mining environments.

### 4.2. Comparison and Summary of Counting Concentration

At low concentrations (0.2~0.3 g/L), the filtration efficiency is higher, especially at low flow rates, with 0.3 g/L showing the best overall performance. At high concentrations (0.4~0.5 g/L), the particle size range expands, leading to reduced efficiency; this is likely related to agglomeration effects and physical detachment. At low flow rates (1.0 m^3^/h), filtration efficiency is highest across all concentrations, particularly for the reduction group’s PM_10_ interception capability. At medium flow rates (1.5 m^3^/h), overall efficiency slightly declines but remains suitable for most concentrations. The 0.3 g/L reduction group demonstrates optimal PM_10_ interception performance. At high flow rates (2.0 m^3^/h), particle concentrations increase significantly (up to 13,000 P/cm^3^), with higher penetration rates for small particles (<1.0 µm), though the 0.3 g/L reduction group maintains good coarse particle interception efficiency. Across all three flow rates, the graphene-modified group performs well, proving that the filtration performance of the composite material after reduction treatment can remain high under light, moderate, and heavy respiratory loads. The performance ranking of the treatment groups is reduction group > oxidation group > control group, with the reduction group exhibiting the lowest particle concentrations and a clear advantage in PM_10_ interception (optimal at 0.3 g/L). Its high-speed resistance surpasses other groups, but its efficiency decreases at high concentrations (0.5 g/L) due to agglomeration. The oxidation group shows moderate filtration efficiency, with a notable rise in small particle concentrations at high flow rates, likely due to enhanced surface oxidation improving adsorption but reducing mechanical stability. The control group has the highest particle concentrations, particularly in the >1.0 µm size range, as it relies on natural sedimentation, making it most affected by flow rates—a finding largely consistent with gravimetric filtration efficiency test results.

Therefore, as the flow rate increases, the filtration efficiency generally decreases. Low-concentration solutions (0.2 g/L and 0.3 g/L) exhibit significant advantages at low flow rates, with the 0.3 g/L reduction group performing the best. In contrast, high-concentration solutions (0.4 g/L and 0.5 g/L) show a marked rise in particulate concentration at high flow rates, and the expansion of particle size ranges along with physical detachment effects also lead to reduced filtration efficiency. Meanwhile, the reduction group demonstrates superior particulate interception performance across all concentrations and flow rates, particularly at low flow rates. Based on a comprehensive comparison of counting efficiency, the 0.3 g/L reduction group is selected as the optimal concentration solution.

Using the two thresholds (efficiency < 95% or resistance > 350 Pa) [37], the estimated service life for the optimal 0.3 g/L reduction group is shown in Table 1.

It is recommended to replace masks after reaching the estimated service life—e.g., heavy labor workers change masks every 2–3 h, while light labor workers change every 8 h. Therefore, with the continuous deepening of underground mines, the application of new mining masks is expected to provide guarantees for the health and safety of personnel in various workplaces [43,44,45,46,47], as well as technical support for the creation of special working environments, thus having greater practical application value.

## 5. Conclusions

This article focused on the issue of insufficient protective performance of mining masks against complex pollution sources in underground metal mines. Through a combination of theoretical analysis, material preparation, and experimental testing, it systematically investigated the filtration performance of novel graphene composite materials and their application effects in mining environments. The main conclusions are as follows:

1. Graphene modification resulted in significantly improved filtration efficiency compared to the control group, with superior reduction treatment effects. The 0.3 g/L reduction group exhibited optimal comprehensive performance: under a flow rate of 1.0 m^3^/h, PM_10_ (95.61%) and PM_2.5_ (95.01%) mass-based filtration efficiency met the standards, while PM_1.0_ (94.88%) approached the standard threshold (difference of only −0.12%), significantly outperforming the control group (PM_10_: 93.39%) and the oxidation group (PM_10_: 95.01%). Moreover, under various flow rates, its particulate concentration was significantly lower than that of the oxidation group and the control group.
2. Optimal performance of the low-concentration graphene solution was observed. In all experiments, the 0.3 g/L reduced group demonstrated relatively stable filtration performance under various gas flow rates, particularly exhibiting superior particle filtration efficiency at medium and low flow rates. The 0.3 g/L reduced group not only exceeded standard requirements in PM_10_ and PM_2.5_ filtration effects but also achieved filtration efficiency close to standard requirements for PM_1.0_, demonstrating highly ideal performance.
3. Count concentration analysis reveals the distribution characteristics of particulate matter, with the reduction group showing significant interception dominance. Fine particles (<1.0 µm) dominate penetration: at a flow rate of 2.0 m^3^/h, the 0.2~0.5 g/L group exhibited over 80% of particles in the 0.265~0.475 µm range, with the reduction group’s concentration significantly lower than that of the oxidation group (approximately 20~30% difference). Large particles (>1.0 µm) demonstrate high interception stability: the reduction group maintains minimal fluctuations in PM_10_ interception efficiency (≤1.3% difference) across the 1.0~2.0 m^3^/h flow range, validating the synergistic effect of mechanical interception and electrostatic adsorption.
4. Based on the experimental data under all flow conditions, the 0.3 g/L reduction group demonstrated the best overall performance, providing relatively ideal filtration effects across different operational intensities (low, medium, and high flow rates). This solution not only meets the filtration requirements for PM_10_ and PM_2.5_ but also exhibits excellent stability and adaptability, making it suitable for use in complex environments such as mines.

## 6. Limitations and Future Research Directions

One of the limitations of this research is that a characterization of material porosity and morphology was not carried out. We also need to factor subjective human evaluations into the performance assessment system for the new mask. To make up for this deficiency, we will conduct in-depth characterization of material microstructure—including porosity and morphology—via SEM and other technologies in subsequent research in order to further explain the intrinsic link between material structure and filtration performance.

## Figures and Tables

**Table 1:** Service life estimation results.

Labor Intensity	Flow Rate (m^3^/h)	Mine Dust Concentration (PM_10_, μg/m^3^)	Estimated Service Life	Limiting Factor
Mild	1.0	100–300	8–12 h	Filtration efficiency decay (drops to <95%)
Moderate	1.5	300–800	4–6 h	Balanced efficiency decay + resistance rise
Severe	2.0	800–2000	2–3 h	Pore blockage (resistance exceeds 350 Pa)

## Data Availability

The original contributions presented in this study are included in the article. Further inquiries can be directed to the corresponding authors.
